# Can extreme experiences enhance creativity? The case of the underwater nightclub

**DOI:** 10.3389/fpsyg.2022.785278

**Published:** 2022-09-27

**Authors:** Daniel C. Richardson, Hosana Tagomori, Joseph T. Devlin

**Affiliations:** Experimental Psychology, University College London, London, United Kingdom

**Keywords:** creativity, divergent thinking, situational effects, cognition, problem solving

## Abstract

Creativity is a valuable commodity. Research has revealed some identifying characteristics of creative people and some of the emotional states that can bring out the most creativity in all of us. It has also been shown that the long-term experience of different cultures and lifestyles that is the result of travel and immigration can also enhance creativity. However, the role of one-off, extreme, or unusual experiences on creativity has not been directly observed before. In part, that may be because, by their very nature, such experiences are very difficult to bring into the laboratory. Here, we brought the tools and empirical methods of the laboratory into the wild, measuring the psychological effects of a unique multisensory experience: an underwater nightclub. We showed – with fully randomized and experimentally controlled conditions – that such an experience boosted measures of divergent thinking in participants. This demonstrates that one element of creativity can be directly enhanced by unusual situations, and that experimental tools of psychology can be used to investigate a range of consumer experiences.

## Introduction

Creativity is of interest to scientists across a range of fields, including the sciences, pedagogy, psychology (Fink and Benedek, 2014), and human resources (Zhou and Shalley, 2011). Creativity is now recognized as central to organizational performance, bringing companies a competitive advantage, and boosting productivity (Serrat, 2017). As such, several businesses have shifted from routinized agendas to those which rely more on knowledge, creativity, and experience (Marjanovic, 2008). However, struggles to maximize the creative potential of employees are still evident (Amabile, 1998; Van Dijk and Van Den Ende, 2002), perhaps due to misconceptions of creativity (Runco and Chand, 1995; Hartley and Greggs, 1997) and a lack of a comprehensive understanding of the factors that contribute to its emergence (Urban, 1991). Increasingly, it is acknowledged that context can exert an influence on the creative process (Plucker et al., 2004; Simonton, 2018).

In this article, we offer novel experimental evidence for such a contextual effect, showing that an unusual experience can cause an increase in creativity. We employed standard laboratory measures of creativity, and deployed them in a non-standard context: an underwater nightclub. This was a unique, multisensory, underwater experience that was part of an experiential marketing event for a drinks brand. We assigned participants at random to our experimental conditions. They underwent a battery of creativity measures immediately after experiencing this underwater nightclub, or in the control group, without having that experience first. Our hypothesis was that the contextual effect of the nightclub would influence creativity. As we review below, while creativity has long been associated with individual differences, more recent research is revealing a systematic effect of contextual effects as well.

### Individual differences and creativity

Most literature has either focused on extrinsic motivators of creativity on an organizational level or employed historiometric methods to analyze creative individuals. Factors such as supervisory and organizational encouragement, autonomy, adequacy of resources, low work pressures, and diversified networks have been previously implicated with enhanced creativity in the workplace (Amabile et al., 1996; Barsh et al., 2008). While this may provide some understanding of the role of social contexts within a firm, measures of creativity in innovation studies are assessed in relative terms on a group output level (Perry-Smith, 2006), thereby failing to reflect the array of complex environments experienced outside workplace settings and its individual contributions.

On the other end of the spectrum, research has long attempted to study individuals whose professions strongly reflect conventional notions of creativity. For instance, Drevdahl and Cattell (1958) sought to explore personality and creativity in eminent artists and writers, and more recently, it was found that psychoactive substances were utilized to facilitate creativity and emotional states amongst artists during the “inspirational” phase of the creative process (Iszaj et al., 2018). However, individuals who are already considered “artists” may vary systematically from the general population, rendering it difficult to achieve generalizable results. Additionally, the direction of causality cannot be established; it is not known whether certain professions lead to creativity or whether more creative people are drawn to those professions. We argue that contextual and individual factors need to be considered in tandem using a diverse sample for a holistic understanding of the antecedents to creativity.

### Contextual effects on creativity

Recent results suggest that diversifying experiences could have beneficial impacts on creativity. Life histories of creative individuals have unearthed common themes of surprise, such as the loss of a parent (Martindale, 1972), developmental adversity (Damian and Simonton, 2015), or having an immigrant status (Goertzel et al., 1978). Furthermore, the eminent scientists Freeman Dyson and Henri Poincare made great discoveries during their travels, while the artist Ernest Hemingway created his most admired pieces following his expatriate experience (de Bloom et al., 2014). However, research on the role of diversifying experiences is still relatively new (Ritter et al., 2012).

Diversifying experiences are highly unusual, unexpected events which are actively experienced by individuals (Ritter et al., 2012), compelling them to embrace novel perspectives, values, and ideas through the breaking of routines (Gocłowska et al., 2018). One example is recreational travel, where well-established cognitive schemas are violated (e.g., through speaking foreign languages), thus increasing the availability of elements for the establishment of associations (de Bloom et al., 2014). Longitudinal studies have shed light on the beneficial role of recreational activities on creativity levels; for instance, it was found that cognitive flexibility (i.e., the ability to deviate from regular cognitive patterns, overcome functional fixedness, and make novel associations; Guilford, 1967) was enhanced in employees following their vacation, even when accounting for workload, vacation hassles, and holiday destinations (de Bloom et al., 2014). Likewise, Maddux and Galinsky (2009) found a positive correlation between years spent abroad and creativity levels on a range of creative tasks (e.g., picture drawing and negotiation activities). This relationship has been elucidated by the Broaden-and-Build Theory, which states that diversifying experiences can lead to positive emotions such as optimism, freedom, and cheerfulness (Chen et al., 2013), expanding their scope of attention and cognition, which, in turn, enhances creative thinking (Fredrickson, 2001). Nonetheless, measuring the number of years spent abroad does not give insight into what aspects of recreational activities modulate creativity.

Snapshot studies examining specific kinds of diversifying interactions have been scarce. The few studies that have been conducted have generated a deviation from familiarity by either violating laws of physics with virtual reality or inducing schema violations through the assembly of a sandwich in a non-conventional order (Ritter et al., 2012). Interestingly, it was found that both these complex and simple violations led to an increase in cognitive flexibility as indicated by a broader range of categories in participants’ responses. Likewise, Leung and Chiu (2010) discovered that the presentation of contrasting Chinese and American cultures or a fusion of the two was associated with an increase in creativity on writing activities than those shown only one culture. However, these experimental manipulations are highly artificial and unlikely to be encountered in the real-world. To the best of our knowledge, the only naturalistic snapshot study was provided by Maddux and Galinsky (2009), who found no significant relationship between temporary vacations and creativity, although it was contended that years after repatriation (a significant factor contributing to the salience of the experience; Maddux and Galinsky, 2009) was overlooked. Furthermore, due to the correlational nature of the study, the types of multicultural experiences could not be isolated. The present study aimed to employ high ecological validity by employing natural settings and using standardized experimental methods to quantify levels of creativity following the experience.

Recently, evidence has built that particular, one-off experiences can influence creative thinking. Chirico et al. (2018) induced a feeling of awe in participants by immersing them in a 3D virtual reality experience, and found an increase in their creativity scores compared to a control group. Similarly, Rastelli et al. (2022) induced a dream-like psychedelic state using VR and found that it increased cognitive flexibility. And more prosaically, but no less impressively, Vohs et al. (2013) showed that the experience of a cluttered disordered room increased the creativity of their participants. Before describing the unusual experience that we induced in our participants, we will review the ways in which creativity can be operationalized and measured.

### Operationalizing creativity

How can creativity be operationalized and measured? Despite variations in the definitions of creativity (Amabile et al., 1996) there has been some consensus that elements of novelty and usefulness form its foundation (Mumford, 2003). It is now acknowledged that the creative process consists of two distinct, yet equally important subprocesses: convergent and divergent thinking (Cropley, 2000, 2006).

The process of convergent thinking is characterized by the ability to discover a single, optimal solution, known to be either valid or invalid (Runco and Acar, 2012), utilizing methods of logical search, evaluative decision-making, and the recognition of conventional rules (Guilford, 1967). One test that measures this process is the remote associates test (RAT), calling for the derivation of a single word which captures the link between a set of word triads by either being synonymous, semantically associated, or forming suitable compounds (Mednick, 1962). For example, given the words “swiss,” “cottage,” and “cake,” the correct answer would be “cheese” as this could precede or follow each of the words in the triad to form a compound. Though there is recent debate in the field over the validity of the RAT as a measure of creativity, it is an extensively used paradigm in the literature, and so we employed it here.

In contrast to convergent thinking, divergent thinking enables several solutions to be generated from an open-ended problem in a less restrictive manner (Colzato et al., 2013) and is key to improvisation and problem solving (Lewis and Lovatt, 2013). A prime example of divergent thinking is the alternative uses task (AUT; Guilford, 1967), whereas many possible uses for familiar objects (e.g., a brick) must be generated within a time limit. Scores are then computed depending on the number of responses (fluency), ability to shift between conceptual categories (flexibility), level of detail (elaboration), and the degree to which responses deviate from the group average (originality). Divergent thinking scores have previously achieved higher correlations with creativity outside experimental settings than convergent tasks; for example, the ability to direct performances, found businesses, and gain patents (Gilhooly et al., 2007).

### Creativity in the context of an underwater nightclub

**Figure 1:**
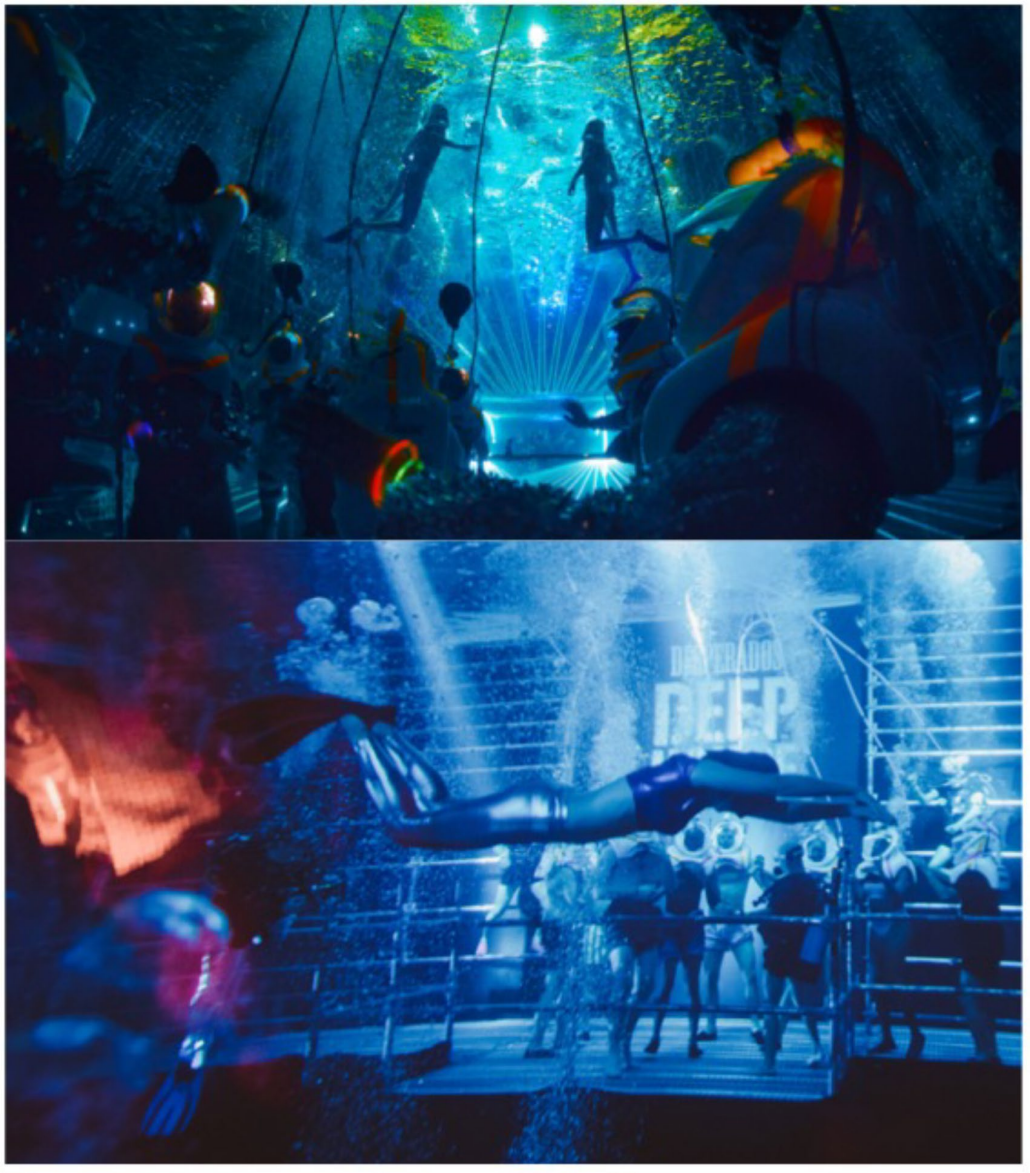
Images of the underwater nightclub experience.

The present study explored how unusual or extreme experiences affect measures of convergent and divergent thinking in a naturalistic setting. We took advantage of a unique, multisensory, underwater experience. Participants wore helmets that were specialized for deep water diving, and dropped to the bottom of a large, specially built pool. A famous club DJ, stationed in a glass tunnel running through the pool, played music that was pumped into the helmets. Participants floated in the pool surrounded by laser lights and divers dressed as mermaids. Figure 1 shows some images from the event.

**Figure 2:**
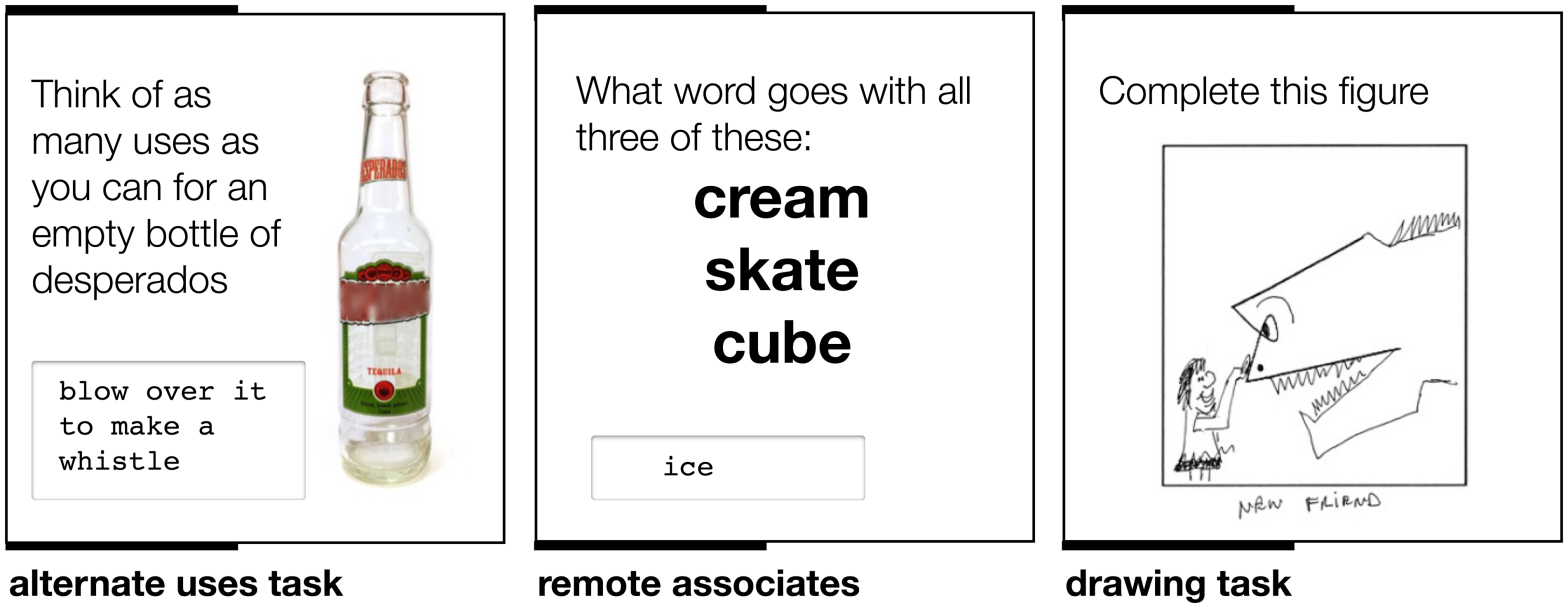
Schematic of creativity task, with example responses.

The participants completed a battery of tasks measuring creativity and mood (Figure 2). Participants were randomly assigned to either the experience group, and were tested immediately after the underwater experience, or to the control condition, and were tested without having experienced the underwater nightclub. Though the event was linked to Desperados, a brand of beer, we were able to ensure that no participants had consumed alcohol prior to being tested. The diving equipment and water depth meant that any alcohol consumption would have been a health risk, and so all participants had to sign a declaration that they had not consumed any at the time of testing. Our measures were able to focus on the effect of the unusual experience alone, and we predicted that individuals in the *experience* group would exhibit greater levels of creativity than individuals in the control group.

## Materials and methods

### Participants

A self-selected sample of 80 participants were recruited through emailed advertisements. Of those, 12 did not take part in the testing on the day for various reasons, such as declining to take part in the underwater experience, or not attending the testing sessions. This left 68 participants in the experiment (29 males, 39 females; age *M* = 26.65 years; *SD* = 4.76). The study was described as a “2-day multisensory experience” involving a dance floor, advanced sound systems, and laser light shows at Deep House, an underwater pool party organized by Desperados, a brand of beer flavored with tequila and sold by Heineken. No monetary compensation was received; however, the costs of transport, accommodation, and party admission were covered by the Desperados team as part of the experiential marketing event.

All participants were native speakers of either English or French and resided in a range of countries: United Kingdom (21%), Italy (18%), Belgium (16%), France (13%), Germany (12%), Netherlands (10%), and others (10%). Approximately, 37% were professionals (e.g., marketing professionals, video producers, and journalists), 16% were students, 12% were entrepreneurs or unemployed, 9% worked in services and sales (e.g., beauticians), 9% as managers (e.g., brand managers), 6% as clerical support workers (e.g., consultants, receptionists), and 12% in other occupations (according to the occupational criteria from the International Labour Organization, 2007). The psychological measures were approved by the University College London Ethics Board.

Participants were randomly and equally assigned the experimental and control conditions. After the participant attrition on the day, we obtained data from 30 in the experience condition and 38 in the control condition. Due to the constraints of managing a live event such as this, some of these participants did not have the time or the drawing materials available when required, and so only 17 participants in the pre group and 24 in the post group were able to do the Incomplete Figures Task.

### Procedure and design

The Deep House party was hosted by Y-40, at the time the world’s deepest indoor pool, located in Venice, Italy. Using a between-groups design, participants were randomly assigned to the experience or control conditions ahead of time. Respondents in the control condition completed a battery of questionnaires and measures of creativity prior to Deep House, and testing took place in a private area of the hotel where participants were lodging. Those in the experience condition completed all tasks immediately after their time in the underwater pool. Testing took place in a cordoned-off corner of the nightclub after they had dried themselves, and before they re-joined the party.

Test administration was divided into two parts: (1) computerized versions of the Brief Mood Introspection Scale (BMIS; Mayer and Gashke, 1988), AUT (Guilford, 1967), Remote Associates Task (RAT; Mednick, 1962), a reduced version of the Ten Item Personality Inventory (TIPI; Gosling et al., 2003) containing only items related to extraversion and openness to experience, and Oregon Research Institute-International Personality Item Pool (ORI-IPIP), for which participants were tested individually in a private room and (2) a paper-and-pencil version of the Incomplete Figures Task. All tasks conducted on the computer were administered using the experimenter builder software Gorilla^1^ (Anwyl-Irvine et al., 2020) and no breaks were present in between each test.

Prior to the task, participants were provided details about the study and the anonymity of their responses was guaranteed. On-site facilitators explained that they were present to assist with technical aspects but that all instructions pertaining to the tasks would be displayed on the tablets or laptops. Following the provision of consent and basic demographic details, participants began the tasks. Once all the required data had been collected, participants were debriefed and thanked for their participation.

### Measures

#### Brief mood introspection scale

An adapted and reduced 9-item version of the original Brief Mood Introspection Scale (BMIS; Mayer and Gashke, 1988) was utilized. Participants were asked to indicate their mood on a 4-point Likert scale (1 = minimum, 4 = maximum) on seven of the original adjectives (i.e., “lively,” “full of ideas,” “tired,” “sad,” ‘calm,” “nervous” and “happy”), with the inclusion of two additional descriptors (“open-minded” and “creative”). From this, scores on three dimensions of mood (positivity, creativity, and activeness) were obtained.

#### Alternative uses task

Based on Guilford (1967) AUT, participants were required to list as many different uses of three common objects (“loudspeaker,” “vinyl record,” and “a bottle of Desperados”) that appeared on a screen within a 45 s time span. All objects were presented sequentially in both pictorial and written form. Responses were typed into a provided space and submitted upon pressing the “enter” key. A countdown timer at the bottom of the screen indicated the final 10 s and participants were notified once the time limit had elapsed. The “continue” button was clicked to proceed to the next object when ready. Typically, the AUT is scored on the frequency, uniqueness, and elaboration of the answers. Our coding scheme had to be automated, so we simplified the process by just counting the number of responses. Though this resulted in a reduced measure of divergent thinking, we employed convergent measures of creativity in the experiment.

#### Remote associates test

In the RAT (Mednick, 1962), participants were presented with three items and asked to think of a fourth related word that could precede or follow each item. As a measure of verbal insight, this requires associations to be identified through the retrieval and reorganization of loosely connected information in memory (Kleibeuker et al., 2013). Prior to task commencement, three exemplar items (“cream,” “skate,” and “water”) and a valid answer “ice” (i.e., “ice cream,” “ice skate,” and “ice water”) were presented in order to ensure participants’ understanding of the task instructions. Respondents were informed that they will have 10 s to generate answers and that a timer will count down the final 5 s. The “enter” key was pressed in order to submit each answer. A total of 10 word-triads were borrowed from Bowden and Jung-Beeman (2003). Test items were presented in a random order for counterbalancing and scores were determined by computing the frequency of all valid answers.

#### Incomplete figures task

A simplified version of the Test for Creative Thinking-Drawing Production (TCT-DP; Jellen and Urban, 1986) was administered. Each participant was presented an irregular, non-geometric, incomplete shape (e.g., a curved line) within a square boundary which was used as a basis for a new drawing under a 5-min time limit. All names were pseudonymized in order to eliminate potential experimenter biases. Drawings were blindly coded by two independent coders, yielding an inter-rater reliability of 0.9. The coders rated the extent to which individuals approached the lines from a new perspective and/or elicited emotion; the extent to which individuals’ drawings deviated from that of other participants; and the extent to which surrealistic, fictional, and abstract elements were included. These scores were combined into a single creativity rating.

## Results

**Figure 3:**
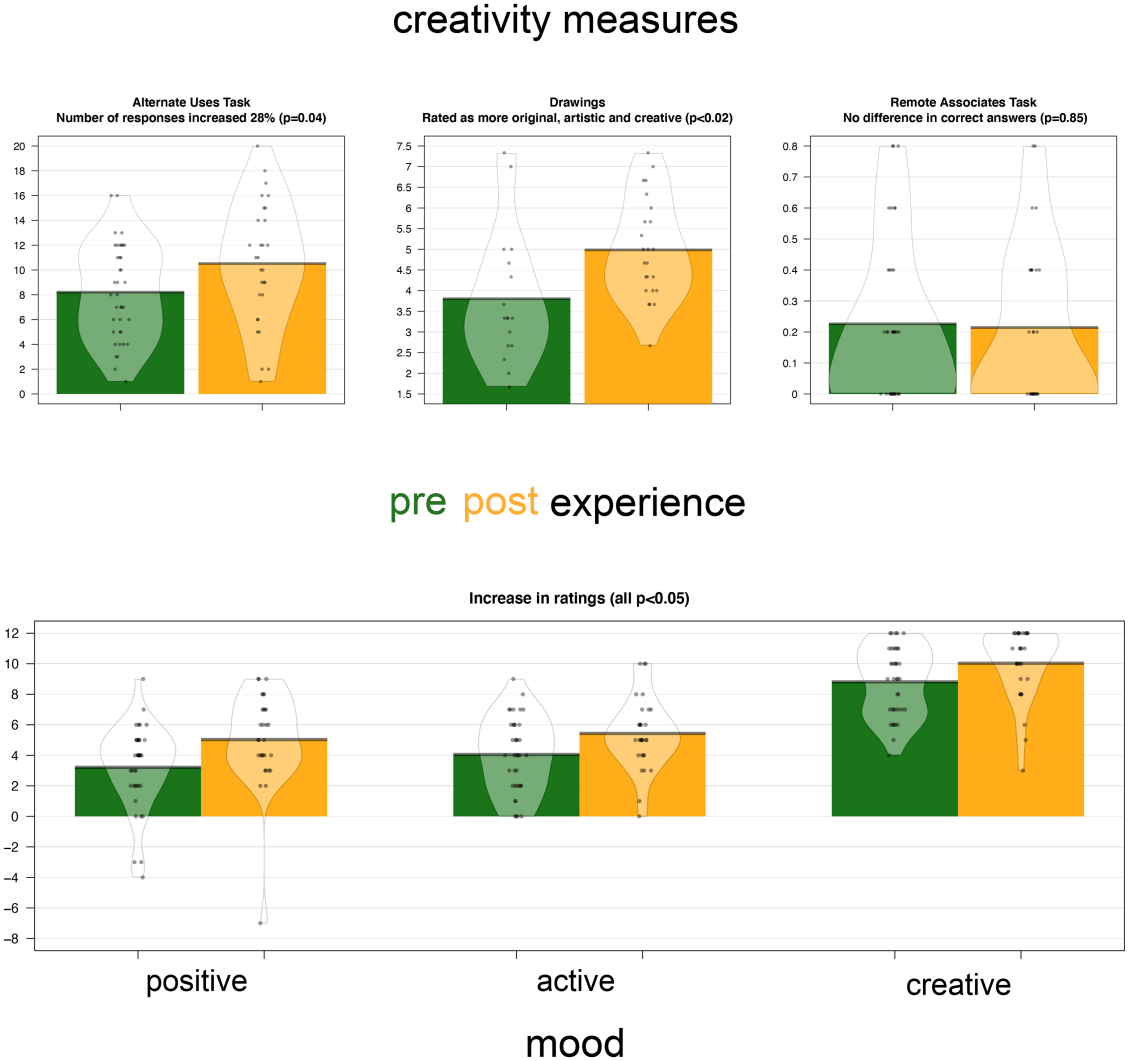
Creativity and mood measures, measure before and after underwater night club experience.

Participants who were tested after their underwater experience scored higher on both measures of divergent creativity (Figure 3, top) and mood (Figure 3, bottom). The effect sizes are all greater than 0.5 suggesting medium or larger sized effects. Table 1 gives a summary of the means, standard deviations and effect sizes for all measures and individual scores across the two conditions. There were no significant differences between the groups on measures of convergent creativity, or on personality scores. Results were analyzed in R (v 3.5.3; R Core Team, 2018). Differences between *experience* and *control* groups were analyzed using Welch’s t-test since in some cases there were unequal cell sizes between conditions.

**Table 1:** Condition means for individual differences, task performance, and mood measures.

Measure Mean (SD)	Experience	Control	*p* value	Cohen’s *d*
Openness to experience (TIPI)	11.9 (2.4)	11.5 (1.89)	0.43	0.19
Extraversion (TIPI)	10.8 (2.75)	9.9 (2.49)	0.16	0.34
ORI	31.4 (5.04)	30.2 (5.79)	0.38	0.22
Remote associations (accuracy)	21.43 (0.26)	22.63 (0.26)	0.85	−0.05
Alternate uses (frequency)	10.5 (4.93)	8.2 (3.86)	0.04	0.51
Drawing rating	4.99 (1.22)	3.80 (1.59)	0.01	0.82
Positive mood (α =0.57)	5 (3.1)	3.2 (2.75)	0.01	0.59
Active mood (α =0.57)	5.4 (2.36)	4.1 (2.44)	0.02	0.52
Creative mood (α =0.84)	10 (2.33)	8.8 (2.26)	0.03	0.51

On the AUT, participants in the *experience* condition produced 28% more suggestions than the *control* group [*t*(51.8) = 2.08, *p* = 0.04]. Coders blind to condition rated the incomplete figure drawings as 31% more creative [*t*(38.6) = 2.57, *p* = 0.016] when they were completed in the *experience* group compared to the *control*. However, accuracy on the remote associate test was not significantly different [*t*(58.2) = 0.19, *p* = 0.85] between conditions, with near equal scores in the *control* and *experience* groups. Following the underwater experience, participants reported elevated mood, with higher ratings for positivity [*t*(58.5) = 2.52, *p* = 0.01], creativity [*t*(61.6) = 2.17, *p* = 0.03], and activeness [*t*(63.3) = 2.36, *p* = 0.02] in the *experience* group.

We carried out a mediation analysis to test if the differences in divergent creativity were due to differences in mood produced by the underwater experience. First, we computed a single variable reflecting divergent creativity by z-scoring and then averaging participants’ scores on the alternative uses and drawing tasks. Using this as the dependent variable, we tested whether each of the mood variables was a significant mediator, using the meditation package (Tingley et al., 2014) that follows Preacher and Hayes (2004). For each of the mood variables, we computed their average causal mediation effects on the creativity score. However, this analysis suggested that neither positive mood (*p* = 0.11), creative mood (*p* = 0.54) nor active mood (*p* = 0.64) scores had a significant mediating effect on creativity.

## Discussion

Most psychology experiments do not take place underwater, surrounded by body glitter, laser lights, and rave music. However, many people seek out precisely such otherworldly experiences, in part due hedonism, a love of music, and the promise of escapism. Without carrying out psychology experiments in these environments, it might not have been known that they have an additional benefit: an increase in creativity. We showed that measures of divergent thinking specifically were enhanced by an underwater nightclub experience.

The question of whether convergent and divergent thinking processes are interdependent or dissociable remains equivocal. A two-step process of creativity where divergent thinking is involved in the initial generation of novelty and convergent thinking in the later evaluation of the effectiveness of ideas has been proposed (Runco, 2003). While Aberg et al. (2017) supported this view through the discovery that those with lower associative processing constraints (lower dopamine levels) demonstrated high scores on both the AUT and the RAT, others have suggested that the two processes are more dissociable than they are interdependent, in terms of EEG alpha wave activities (Jauk et al., 2012) and the extent to which they rely on top-down executive control (Colzato et al., 2013). These differences have also translated behaviorally; risk taking has been negatively associated with convergent but not divergent thinking (Shen et al., 2018). Our results suggest that diversifying experiences have a specific effect on the divergent thinking aspect of creativity.

We found that participants’ mood was positively enhanced by their experiences underwater. Previous research has found that affect plays a role in creativity. For example, Isen et al. (1987) induced positive emotions through depicting humor in films and offering candy to participants, finding a subsequent rise in creativity scores. Similar results have been observed in a sample of physicians after reading statements conveying practice satisfaction (Estrada et al., 1994), and on an array of creative tasks (e.g., grouping objects: Isen and Daubman, 1984; bargaining exercises: Carnevale and Isen, 1986). Isen (1999) explained this as a result of a defocused attention, augmenting the availability of cognitive elements and flexibility. Though we found that mood was elevated, we did not see significant evidence that mood by itself explained the increase in creativity that we saw after the underwater nightclub experience.

Were our results due to the particular type of person who took part in this experience? It is true that certain dimensions of personality have been linked with creativity, namely openness to experience and extraversion, which have been thought to involve the cerebellum, an area of the brain implicated with task switching and adaptation (Feist, 2019). Individuals who score highly on openness to experience are highly motivated to seek out novel experiences and perspectives, are broad-minded, imaginative, curious, and original (McCrae and Costa, 1987). Personality has been discovered to mediate the positive impact that multicultural experiences have on creativity; the effect was more robust in those who identified with their host culture (Tadmor et al., 2009) and exceeded a threshold in their motivation to engage themselves with a new environment (Leung et al., 2008). However, Leung et al. (2008) operationalized openness to experience as the extent to which American undergraduates sampled ideas from foreign scholars, which imposes an issue as this is confined to a highly specific context; therefore, the current study would ensure a greater applicability of this dimension across a range of situations.

Since our conditions were randomized, and we found no significant differences between groups on our personality measures, we can be confident that these results were not due to particular characteristics of the participants involved. Since we can be sure that the participants had not yet indulged in alcohol consumption, we can be confident that it was specifically the other-worldly aspect of the experience that had a psychological effect. This result is in line with previous work showing that the experience of travel and other cultures can increase creativity.

## Conclusion

We argue that our experiment has some value as a real-world test of the effects of diversifying experiences on creativity. But, as ever, that ecological validity comes at a cost to the precision and generalizability of our claims. We do not know, for example, which elements of the underwater nightclub – the music, the weightless swimming, changes in blood oxygen levels, the disorientating lasers – were responsible for the shifts in creativity. Or indeed, whether people in the pre-condition, waiting at the hotel, perhaps feeling bored or apprehensive, had relatively depressed levels of creativity. We would argue, however, that in the context of other more controlled lab studies on creativity, this experiment points toward the unusual elements of the experience as having a positive effect on creativity. Moreover, we argue that it expands the psychological literature by showing that a single night of an intense and unusual experience can have the same effect. And perhaps provides some motivation for psychologists, and others, to seek out experiences outside of the everyday.

## Data availability statement

The raw data supporting the conclusions of this article will be made available by the authors, without undue reservation.

## Ethics statement

The psychological measures were approved by the University College London Ethics Board. The patients/participants provided their written informed consent to participate in this study.

## Author contributions

DR and JD designed the experiment. DR collected and analyzed data. All authors contributed to the article and approved the submitted version.

## Conflict of interest

The underwater experience described in the MS was part of an experiential marketing event for Desperados, a brand sold by Heineken, who funded the event. One author was hired as a consultant to design and administer an experiment investigating the psychological consequences of diversifying experiences. Heineken played no role in designing the experiment or analyzing data.

## Publisher’s note

All claims expressed in this article are solely those of the authors and do not necessarily represent those of their affiliated organizations, or those of the publisher, the editors and the reviewers. Any product that may be evaluated in this article, or claim that may be made by its manufacturer, is not guaranteed or endorsed by the publisher.

## Footnotes

^1^ https://gorilla.sc
